# Projections of temperature and precipitation changes in Xinjiang from 2021 to 2050 based on the CMIP6 model

**DOI:** 10.1371/journal.pone.0307911

**Published:** 2024-10-09

**Authors:** Yunlei Zhang, Pei Zhang, Xinchen Gu, Aihua Long

**Affiliations:** 1 College of Management and Economics, Tianjin University, Tianjin, China; 2 Institute of Water Resources and Hydropower Research, Beijing, China; 3 State Key Laboratory of Hydraulic Engineering Simulation and Safety, School of Civil Engineering, Tianjin University, Tianjin, China; Arab Academy for Science Technology and Maritime Transport, EGYPT

## Abstract

Xinjiang is one of the most sensitive regions in China in terms of its response to climate change. Against the background of global warming, analyses and predictions using different scenarios for Xinjiang should be conducted. The spatial and temporal distribution characteristics and trends of future temperature and precipitation trends should be considered to provide a scientific basis for the government to respond to future climate change. In this paper, using the CN05.1 dataset and seven models from the sixth phase of the Coupled Model Intercomparison Project, the delta downscaling method is used to predict the temperature and precipitation changes in Xinjiang Province from 2021 to 2050 under the SSP1-2.6, SSP2-4.5, SSP3-7.0, and SSP5-8.5 scenarios. The results show that (1) most models of CMIP6 have a good effect on temperature simulation in Xinjiang, and the mean values as well as the trends of the temperatures expressed by the multi-model ensemble averaging are in good agreement with the observed data and have a high degree of confidence. The observed precipitation increase rate is significantly higher than that predicted by the model, and the simulation results of each model overestimate the precipitation. (2) The mean annual temperatures in the Xinjiang region increase at rates of 0.32°C/10 a, 0.46°C/10 a, 0.47°C/10 a and 0.67°C/10 a, respectively, under the four scenarios. The rates of temperature increase in the four seasons exhibit the following pattern: autumn > summer > spring > winter. (3) From 2021 to 2050, the average annual precipitation in Xinjiang will change at rates of 3.95 mm/10 a, 1.90 mm/10 a, 2.50 mm/10 a, and 8.67 mm/10 a, respectively, under the four scenarios. The precipitation amounts predicted under the different scenarios increase at the slowest rates in winter and at faster rates in spring. Spatially, the precipitation in the whole Xinjiang region under the four scenarios shows an increasing trend. Overall, except for the SSP1-2.6 scenario, the rates of increase in precipitation increase gradually in all seasons during the future period as the emission scenarios increase. Overall, the climate of the Xinjiang region will be characterized by warming and humidification from 2021 to 2050.

## 1. Introduction

In 2021, the Fifth Assessment Report of the United Nations Intergovernmental Panel on Climate Change (IPCC) stated that, using the 1986–2005 global multiyear average temperature as a baseline value, the mid-century temperatures may warm by approximately 0.3–0.7°C, and the temperatures at the end of the century may warm by approximately 4.5°C. Continued global warming has had serious impacts on world development, not only by altering the duration of each year during which vegetation and crops need water and exacerbating the tendency toward drought within a given period but also by altering the characteristics of the regional hydrological cycle, exacerbating the uncertainty in water resources, and increasing the frequency and intensity of undesirable meteorological hazards, such as droughts.

The Coupled Model International Comparison Project (CMIP) contains many global climate models (GCMs), which provide a data basis for climate modeling and future climate change projections and related research [1]. Over the past two decades, great progress has been made in global climate models. In 1995, CMIP1 [2] was first introduced with only 10 models; by the time CMIP5 was introduced, more than 40 climate models were available [3], but CMIP5 currently has some limitations in different scenarios. Compared to CMIP6, CMIP5 includes fewer models, designs fewer numerical experiments, and provides less simulation data. Moreover, the ability of the CMIP5 model to simulate spatial temperature and precipitation distributions in China is inferior to that of the CMIP6 model. CMIP6 improves the resolution of the models, which is closer to reality, and provides a multifaceted perspective on the Earth’s system from multiple perspectives [4]. CMIP6 proposes eight new climate projection scenarios, which are rectangular combinations of different SSPs and RCPs. Shared socioeconomic pathways (SSPs) are shared socioeconomic pathways that assume the possible future development of society in the absence of climate policies or climate change impacts. Representative concentration pathways (RCPs) are typical emission pathways and include RCP2.6, RCP4.5, RCP6.0 and RCP8.5. These results indicate that by 2100, the radiative forcing levels will be stable at approximately 2.6, 4.5, 6.0 and 8.5 W/m^2^, respectively. The SSP1-2.6 scenario shows the combined effects of low radiative forcing, low vulnerability and low mitigation pressure, while the SSP2-4.5, SSP3-7.0 and SSP5-8.5 scenarios indicate moderate, medium-to-high and high levels of development, respectively, with moderate, relatively high and high vulnerability, respectively.

GCM climate models have low resolution, and their direct use will produce large biases when predicting future climate trends [5,6]. Downscaling methods can transform large-scale coarse resolution information into a fine grid with high spatial resolution. Downscaling methods include statistical downscaling and dynamical downscaling, in which the physical significance of dynamical downscaling is clear but the computational volume is large and the simulation configuration is inconvenient; moreover, statistical downscaling has a small computational volume, the model construction is simple, and the simulation effect is also very good [7–9].

With global warming, the temperatures in China have also experienced warming, but the growth rates vary significantly from place to place. Since 1961, the annual mean surface temperature in China has increased significantly, reaching 0.24°C/10 a. Xinjiang is located in the inland part of northwestern China, far from the ocean, where little precipitation occurs and high levels of evaporation are present; this region is a typical arid climate zone and ecologically fragile area. Precipitation mainly originates from westerly circulation and from cold and humid airflow from the Arctic Ocean southward, while it is difficult for the water vapor that is generated by monsoons from both the Pacific and Indian Oceans to reach Xinjiang. In this paper, the possible future changes in temperature and precipitation in Xinjiang, China under different emission scenarios are projected to provide scientific support for a better understanding of the climate change characteristics of Xinjiang.

At present, there have been many domestic and foreign studies on the climate in various regions and in various basins [10–13], but studies on the entire Xinjiang climate using the latest CMIP6 model data are still limited [14,15]. Studying climate change simulations in Xinjiang is very important for understanding its trends and mechanisms. Therefore, in this paper, the temperature and precipitation data from 1961 to 2014 in CN05.1 were used as the observational data, and delta downscaling [16] and deviation corrections were carried out for the temperature and precipitation data in four different climate model scenarios (e.g., SSP1-2.6, SSP2-4.5, SSP3-7.0 and SSP5-8.5) of CMIP6. The multimodel ensemble averages of the temperatures and precipitation amounts in the future period were obtained, and the spatiotemporal variation trends of temperature and precipitation were analyzed by using the ensemble average data. The results of this study have important reference value for determining the response to climate change and for the sustainable development of the economy and agriculture in Xinjiang.

## 2. Data sources and methods

### 2.1. Overview of the study area

**Fig 1:**
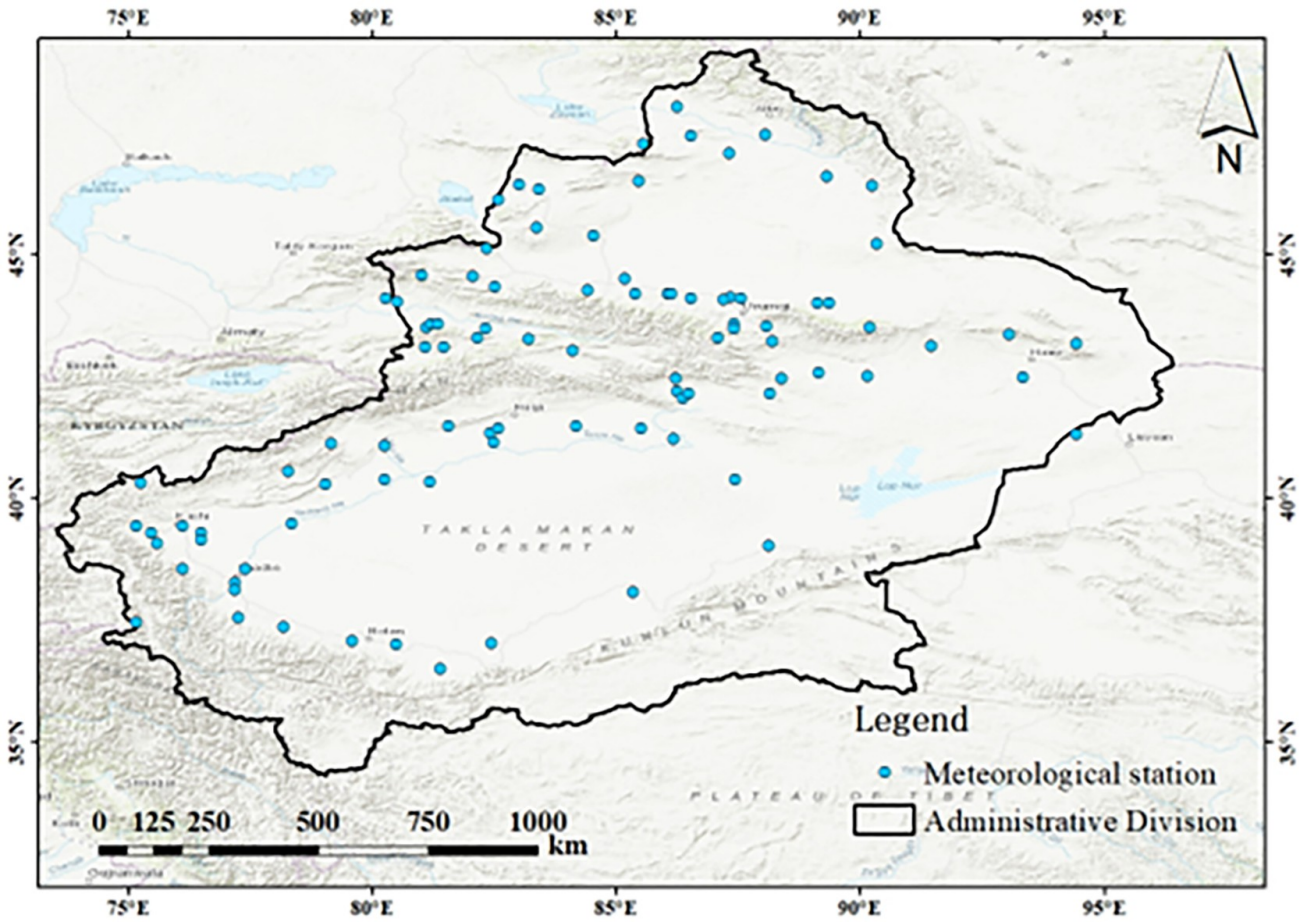
The distribution of the topographic and meteorological stations in the study area.

As shown in Fig 1, Xinjiang is located in the hinterland of the Eurasian continent at middle latitudes, between 73°20’-96°25’E and 34°15’-49°10’N. This region has a total area of 1.66×10^6^ km^2^, which accounts for about one-sixth of the country’s total area, and is the largest provincial-level administrative division in China. Xinjiang has a total of 14 prefecture-level administrative boundaries, including five autonomous prefectures, five regions and four prefecture-level cities [17]. Xinjiang is a typical oasis agricultural region with an extremely fragile ecosystem and is sensitive to climate change.

Xinjiang has a typical temperate continental arid climate with long winters, relatively short spring and autumn periods, many hours of sunshine in summer, and large temperature differences between day and night. The average annual precipitation in Xinjiang is 159.43 mm, the average annual potential evapotranspiration is 1084.28 mm, the average annual temperature is 8.32°C, and the average annual sunshine duration is 2836 h [18]. The precipitation distribution in Xinjiang is extremely uneven. For example, the Junggar Basin is surrounded by the central Tianshan Mountains, the Altar Mountains and the western mountains. As a result, the water vapor that is generated by the westerly flow can reach only the Junggar Basin from the Yili River Valley to the southwest. Due to the obstruction of the Tianshan Mountains, relatively large amounts of precipitation occur only in the Yili River Valley [19]. Moreover, the Tianshan Mountain range, which towers over central Xinjiang, creates distinct climatic characteristics between the northern and southern parts of the region [20]. The spatial and temporal distributions of the water resources in Xinjiang are extremely uneven. With global climate change and human overexploitation of groundwater and other activities, the hydrology, water resources, water ecology and water resources cycle in Xinjiang have changed to a certain extent. In particular, the occurrence of global warming has intensified the instability of Xinjiang’s water resource system and the risk of drought caused by related water scarcity.

### 2.2. Data sources

#### 2.2.1. CN05.1 data

In this paper, the mean temperature and precipitation of the CN05.1 dataset were selected with a resolution of 0.25°. The CN05.1 dataset contains daily observation data obtained by Wu Jia et al. [21] from more than 2,400 national stations of the National Meteorological Information Centre (NMIC); these data were interpolated and superimposed by using the thin-disk spline function method (ANUSPLIN) and the angular distance weighting method (ADW), respectively [22]. All the gridded data files were subjected to strict quality inspection control, data verification, data corrections and supplementation [23] and have been widely recognized and applied in China [24–26].

#### 2.2.2. CMIP6 dataset

In this study, the historical and future data of seven modes of the CMIP6 dataset were adopted with reference to the relevant literature, and the source of the data is the Sixth Coupled Model Intercomparison Project (https://esgf-node.llnl.gov/projects/cmip6/). The detailed information is shown in Table 1.

**Table 1:** Summary of the CMIP6 models selected in this paper.

Number	Model name	Institution	ECS	Spatial resolution
1	ACCESS-ESM1-5	CSIRO-BOM	3.9°C	1.875°×1.25°
2	CanESM5	CCCma	5.6°C	2.8125°×2.8125°
3	CMCC-ESM2	FCE-MCC	3.6°C	1.25°×0.9424°
4	CNRM-ESM2-1	CNRM	4.8°C	1.4063°×1.4063°
5	INM-CM4-8	INM	1.8°C	2°×1.5°
6	INM-CM5-0	INM	1.9°C	2°×1.5°
7	MRI-ESM2-0	MRI	3.2°C	1.125°×1.125°

The CMIP6 model data have a daily temporal resolution. In this paper, the data from the Xinjiang region from 1961 to 2014 were selected to simulate the historical phase, and the future study period runs from 2021 to 2050. To facilitate comparative analyses with climate observations, the resolutions of the seven climate models were resampled to a resolution that is consistent with the observed data using a bilinear interpolation method.

### 2.3. Research methodology

#### 2.3.1. Delta downscaling and bias corrections

The Global Climate Model (GCM) is a numerical model that is widely used to simulate the Earth’s climate system. Global climate models are good predictors of future climate change. Although the physical properties and resolution of the latest generation of global climate models, CMIP6, have improved, global climate models are better matched to observations at large scales than are local regional models [27,28]. Therefore, downscaling and bias corrections of climate models are necessary before carrying out future climate projections [29]. Downscaling is the use of interpolation to resample low-resolution grid information to higher-resolution, applicable regional-scale data [30]. Compared to the kinetic downscaling method, the statistical downscaling method is computationally limited, easy to apply, and widely applicable.

The delta space scaling method Is simple and commonly used. The differences between future and historical periods (such as the absolute increases in temperature and the relative change rates of precipitation) are defined as climate change signals. This signal can be used with site or regional historical observation data to obtain future climate change scenarios [31]. In this study, the delta downscaling method was chosen to downscale the CMIP6 model data to the resolution of the observed data (0.25° × 0.25°). The differences between the resampled model data and the observed data were taken as the deviations between the simulations and observations, and bias corrections were performed for the data from different months at different locations. The calculation formula is as follows:

$$T_{f}=T_{0}+(T_{Mf}-T_{M0})$$ (1)

where *T*_*f*_ is the future grid temperature series that is reconstructed by the delta method, *T*_*Mf*_ is the future simulated grid month-by-month temperature data, *T*_*M*0_ are the simulated grid month-by-month mean temperature data for the base period, and *T*_0_ are the observed multiyear mean temperature data for the base period.

$$P_{f}=P_{0}\times (P_{Mf}/P_{Mo})$$ (2)

where *P*_*f*_ is the future grid precipitation sequence that is reconstructed by the delta method, *P*_*Mf*_ is the simulated grid month-by-month precipitation data for the future period, *P*_*M*0_ is the simulated grid month-by-month average precipitation data for the base period, and *P*_0_ is the measured multiyear average precipitation data for the base period.

#### 2.3.2. Bilinear interpolation

The bilinear interpolation method is simple and is only slightly more complex than the nearest neighbor interpolation method, and the interpolation results are smoother than those provided by other methods [32,33].

The coordinates of the four points, namely, *Q*_11_(*x*_1_, *y*_1_), *Q*_12_(*x*_1_, *y*_2_), *Q*_21_(*x*_2_, *y*_1_), and *Q*_22_(*x*_2_, *y*_2_) of the function f are known, along with the corresponding pixel values. Interpolation is first performed in the x-direction to obtain *R*_1_ and *R*_2_ and then in the y-direction to obtain the pixel values of the function f at the intermediate point P(x,y).

In this case, *R*_1_ and *R*_2_ are calculated as follows:

$$f(R_{1})\approx \frac{x_{2}-x}{x_{2}-x_{1}}f(Q_{11})+\frac{x-x_{1}}{x_{2}-x_{1}}f(Q_{21}),R_{1}=(x,y_{1})$$ (3)

$$f(R_{2})\approx \frac{x_{2}-x}{x_{2}-x_{1}}f(Q_{12})+\frac{x-x_{1}}{x_{2}-x_{1}}f(Q_{22}),R_{2}=(x,y_{2})$$ (4)

Interpolation in the y-direction yields P:

$$f(P)\approx \frac{y_{2}-y}{y_{2}-y_{1}}f(R_{1})+\frac{y-y_{1}}{y_{2}-y_{1}}f(R_{2})$$ (5)

In this study, a Python-based code editor, Jupyter Notebook, was used to perform bilinear interpolation on seven kinds of CMIP6 data to obtain consistent spatial resolution data.

## 3. Results and analyses

When applying future climate data, the downscaling and bias correction processes should be carried out first so that the rough grid becomes a high-resolution fine grid before being applied at a small regional scale. In this section, the temperature and precipitation data from 1961 to 2014 obtained from the CN05.1 observations were first used to evaluate the simulation effect of the climate model in the Xinjiang region. The future model data were used to analyze the interannual and seasonal spatiotemporal characteristics of the temperature and precipitation in the Xinjiang region during the period from 2021 to 2050.

### 3.1. CMIP6 data preprocessing

In this study, the model information (temperature T and precipitation P) from CMIP6 was used to average seven models over the period from 1961 to 2014, and ensemble averaging results were thus obtained to predict the climate change situation in the Xinjiang region.

**Fig 2:**
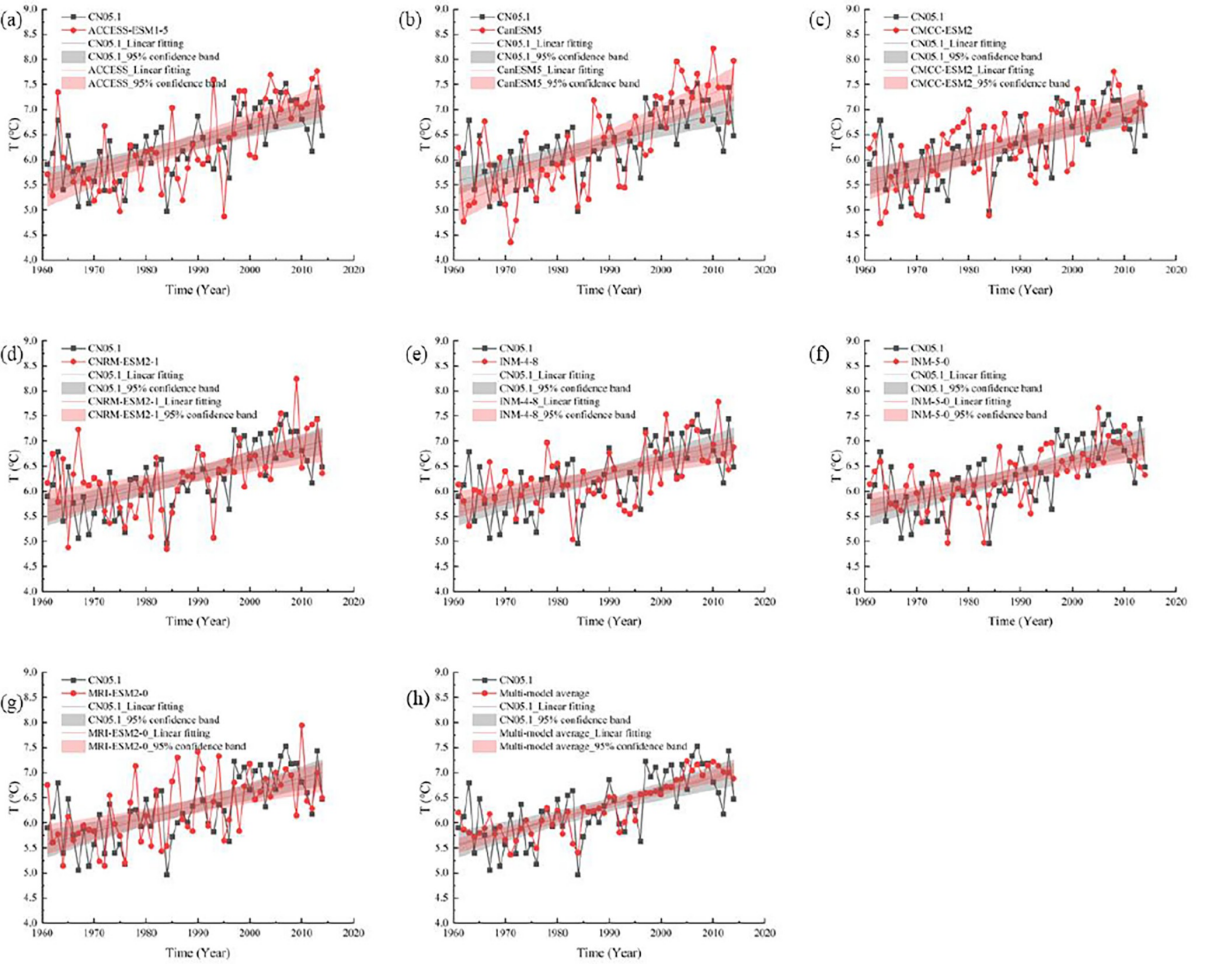
Characteristics of annual mean temperature change in Xinjiang during 1961–2014.

**Fig 3:**
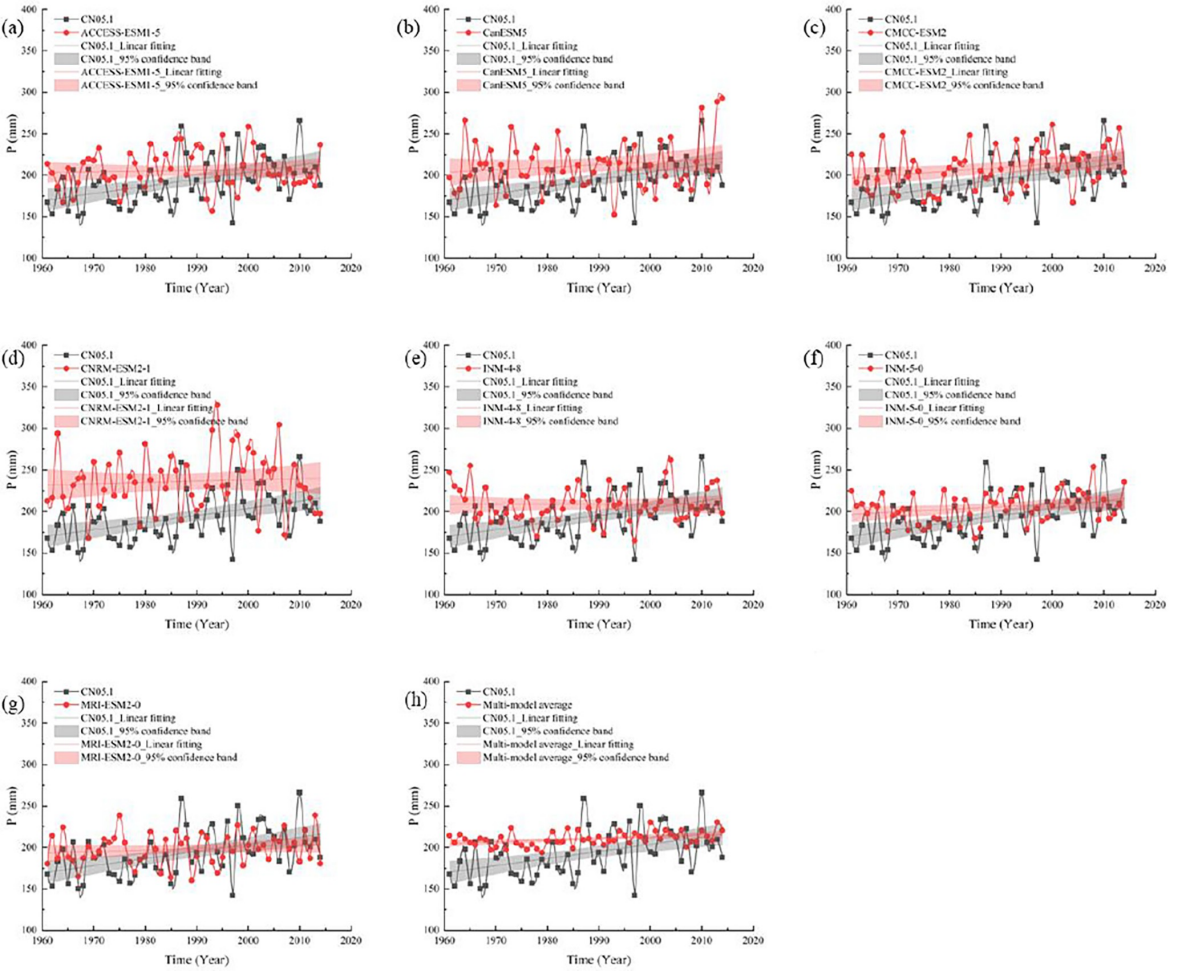
Change characteristics of annual mean precipitation in Xinjiang during 1961–2014.

As shown in Figs 2 and 3, the simulation effects of the seven models on the temperature in Xinjiang are good, and the multimodel average results of the temperature simulations are also in good agreement with the CN05.1 observation data. The downscaling effect of precipitation is not good, which may be caused by the inherent uncertainty of the model or the selection of the unused downscaling method, which may lead to the deviation between the model prediction and the actual data. The multimodel mean precipitation results exhibit small variations and very similar variations across all periods. The multimodel average results are more consistent with the observed data than are those of a single climate dataset and can better reproduce the temporal trends of the observed climate variables.

It has been demonstrated that multimodel climate ensemble data can simulate temperature and precipitation better than single-model simulations and can reduce the uncertainties in future scenarios that are simulated by a single model to some extent [34].

### 3.2. Future temperature predictions in Xinjiang

#### 3.2.1. Trends

**Fig 4:**
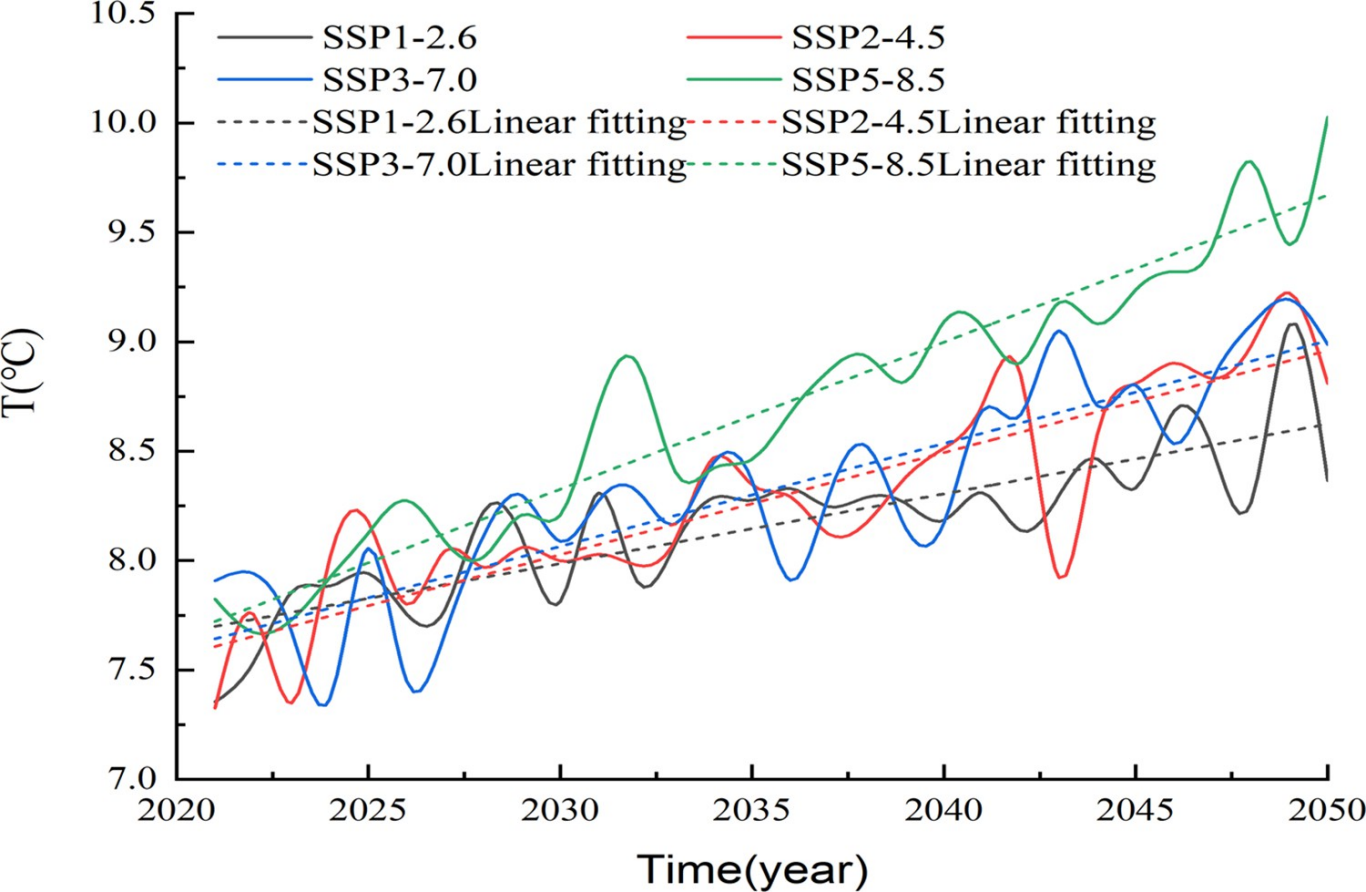
Interannual variation trend of mean temperature in Xinjiang during 2021–2050.

We used temperature data generated by the bias-corrected multimodal ensemble under four scenarios (e.g., SSP1-2.6, SSP2-4.5, SSP3-7.0 and SSP5-8.5) to simulate the temperature changes in Xinjiang from 2021 to 2050. The entire Xinjiang region shows increasing temperature trends for all four SSPs from 2021 to 2050. Under the SSP1-2.6, SSP2-4.5, SSP3-7.0 and SSP5-8.5 scenarios, the annual mean temperatures increased slightly at rates of 0.32°C/10 a, 0.46°C/10 a, 0.47°C/10 a and 0.67°C/10 a, respectively. Fig 4 shows that the temperature differences between the SSP2-4.5 and SSP3-7.0 scenarios are very small from 2021 to 2050. The temperature differences among the four scenarios from 2021 to 2035 are small, but become significant after 2035. In particular, the temperature differences among the SSP5-8.5 scenario and the other three scenarios are large.

**Fig 5:**
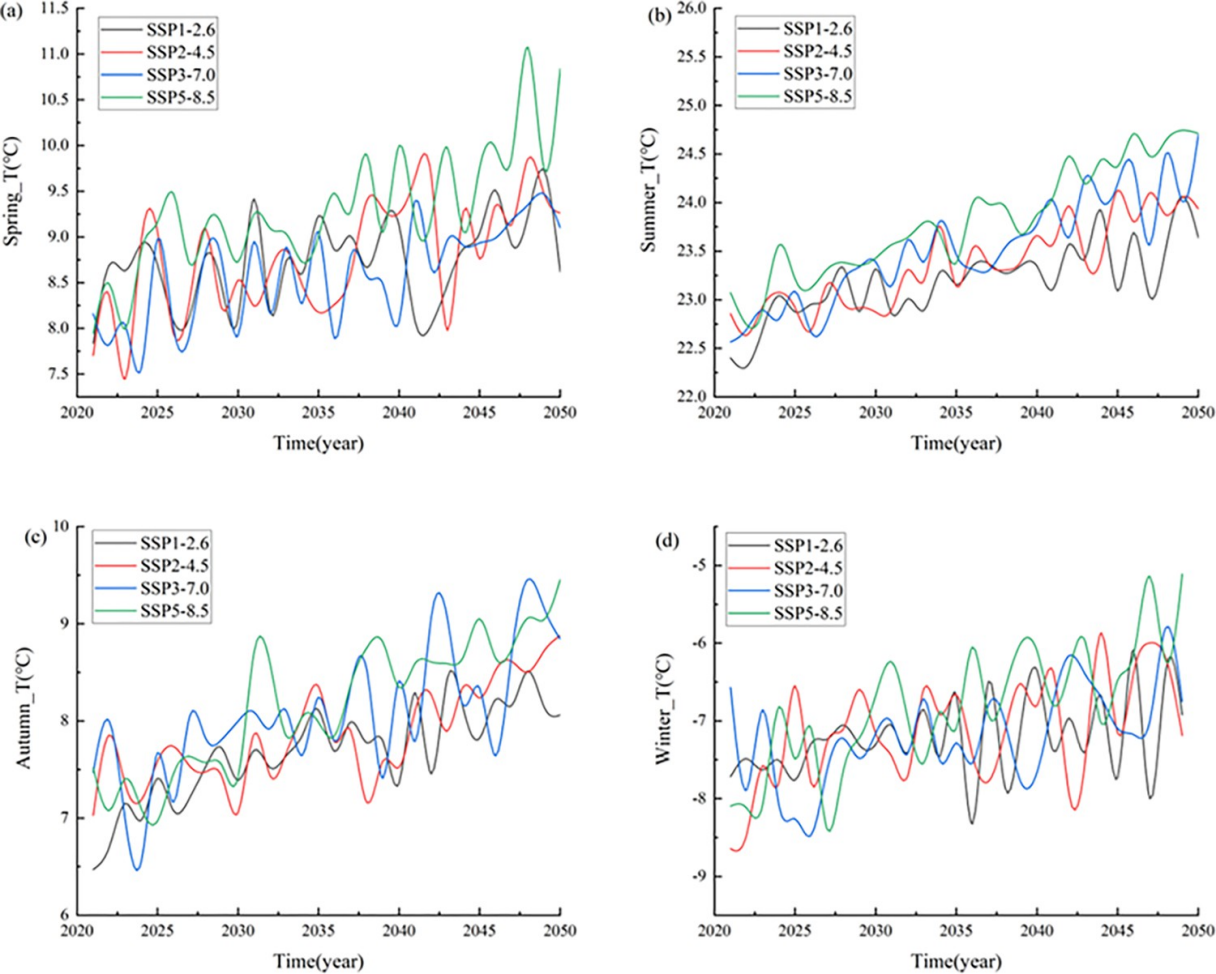
Seasonal variation trend of mean temperature in Xinjiang from 2021 to 2050.

In addition, we analyzed the changes in the seasonal mean temperature in Xinjiang (Fig 5), which showed fluctuating upward trends in all seasons from 2021 to 2050. Overall, the rates of temperature increase for the four seasons exhibited the following pattern: autumn > summer > spring > winter.

The results suggest that the air temperatures in the Xinjiang region in all four seasons show increasing trends under the different SSP scenarios; the higher the radiative forcing is, the greater the warming rate.

In spring, the temperatures increased at rates of 0.24°C/10 a, 0.47°C/10 a, 0.42°C/10 a and 0.62°C/10 a under the SSP1-2.6, SSP2-4.5, SSP3-7.0 and SSP5-8.5 scenarios, respectively. In summer, the climate propensity rates under the four scenarios were 0.35°C/10 a, 0.45°C/10 a, 0.59°C/10 a, and 0.62°C/10 a, respectively (Table 2). In autumn, the temperatures under scenarios SSP1-2.6, SSP2-4.5, SSP3-7.0, and SSP5-8.5 increased at rates of 0.47°C/10 a, 0.46°C/10 a, 0.54°C/10 a and 0.71°C/10 a, respectively, showing continuous increasing trends. In winter, the climate propensity rates of the four scenarios are 0.22°C/10a, 0.46°C/10a, 0.38°C/10a and 0.77°C/10a, respectively. In general, the greater the radiative forcing is, the greater the rate of increase in the seasonal temperature.

**Table 2:** Seasonal and interannual temperature trends under four scenarios in Xinjiang during 2021–2050.

Trend °C/10a	Spring	Summer	Autumn	Winter	Interannual
SSP1-2.6	0.239	0.347	0.473	0.224	0.319
SSP2-4.5	0.465	0.450	0.456	0.461	0.466
SSP3-7.0	0.415	0.586	0.538	0.382	0.470
SSP5-8.5	0.619	0.622	0.705	0.774	0.672

#### 3.2.2. Spatial distribution

**Fig 6:**
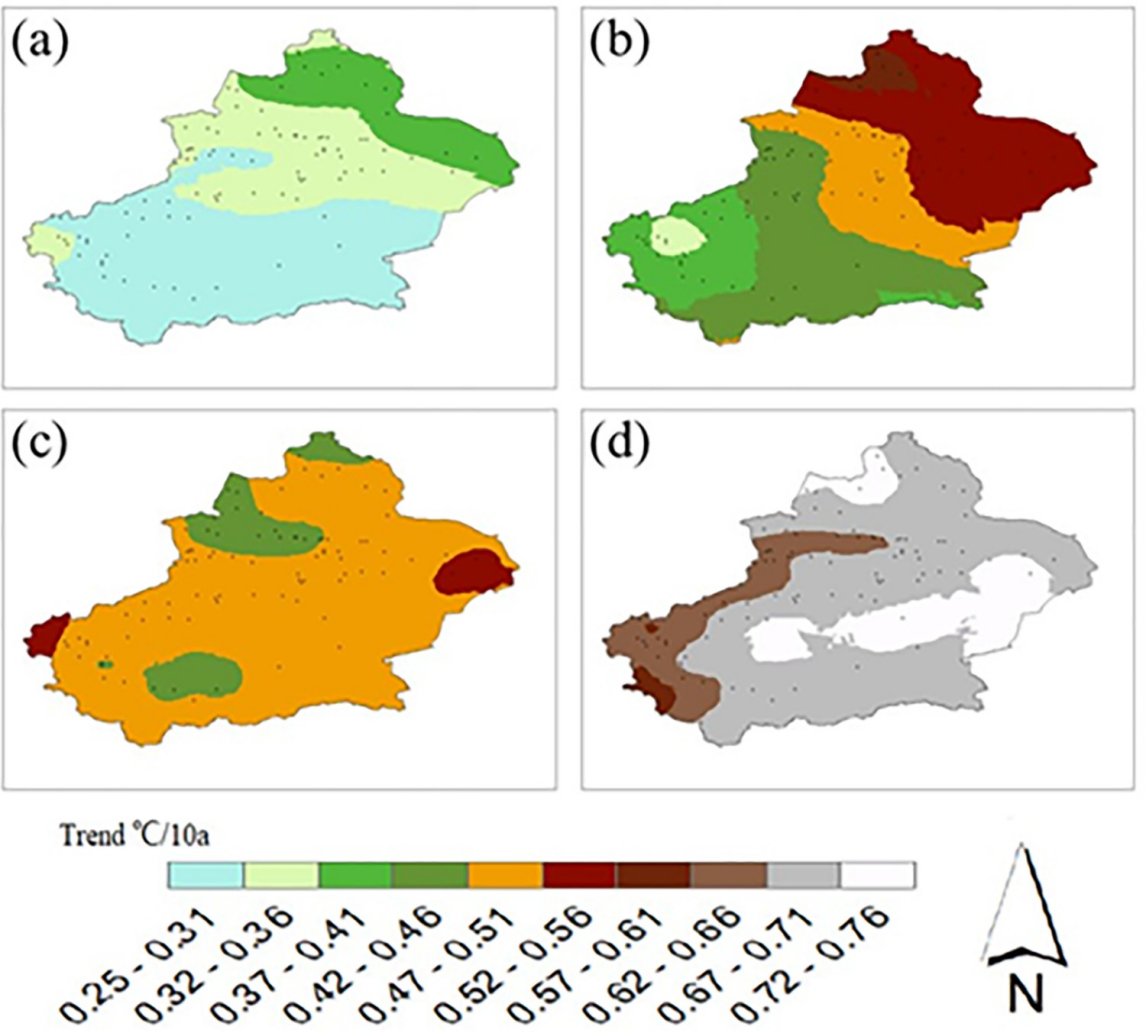
Spatial distribution of future mean temperature trends in Xinjiang. (a)SSP1-2.6、(b)SSP2-4.5、(c)SSP3-7.0、(d)SSP5-8.5.

This study analyzed the spatial distributions of the future (2021–2050) mean annual temperature trends (Fig 6), and there were large spatial differences in the warming trends under different scenarios. The results show that the temperatures in the whole Xinjiang region will increase under all four scenarios, and the trends increase with increasing radiative forcing.

Under the SSP1-2.6 scenario, the warming rates are greater in the northern part of the Altay region and in Barkun and Yiyu counties in the northern part of Hami; additionally, the warming rates are greater in the northern part than in the southern part of the region, and the rates of increase in the mean annual temperatures gradually become greater from south to north.

Under the SSP2-4.5 scenario, the areas with greater warming rates are mainly concentrated in Altay, Hami, Tacheng, and Turpan in Changji, and the rates of increase in the mean annual temperatures gradually become greater from the southwest to the northwest.

Under the SSP3-7.0 scenario, the areas with greater warming rates are mainly located in the eastern part of Hami city and the western part of Kezhou, and the rates of temperature increase in the vast majority of Xinjiang range from 0.47 to 0.51°C/10 a.

Under the SSP5-8.5 scenario, the areas with the highest warming rates are mainly present in the northwestern parts of Tacheng and Altay, the Tarim Basin and the southern part of Hami, and the rates of temperature increase in most of Xinjiang range from 0.67–0.72°C/10 a.

Comparing the four scenarios, the smallest warming trend in Xinjiang occurs under the SSP1-2.6 scenario, a smaller warming trend occurs under the SSP2-4.5 scenario and the smaller differences in the warming trends across Xinjiang; the warming trends in the SSP3-7.0 scenario are a bit larger; and the largest warming trends are under the SSP5-8.5 scenario.

**Fig 7:**
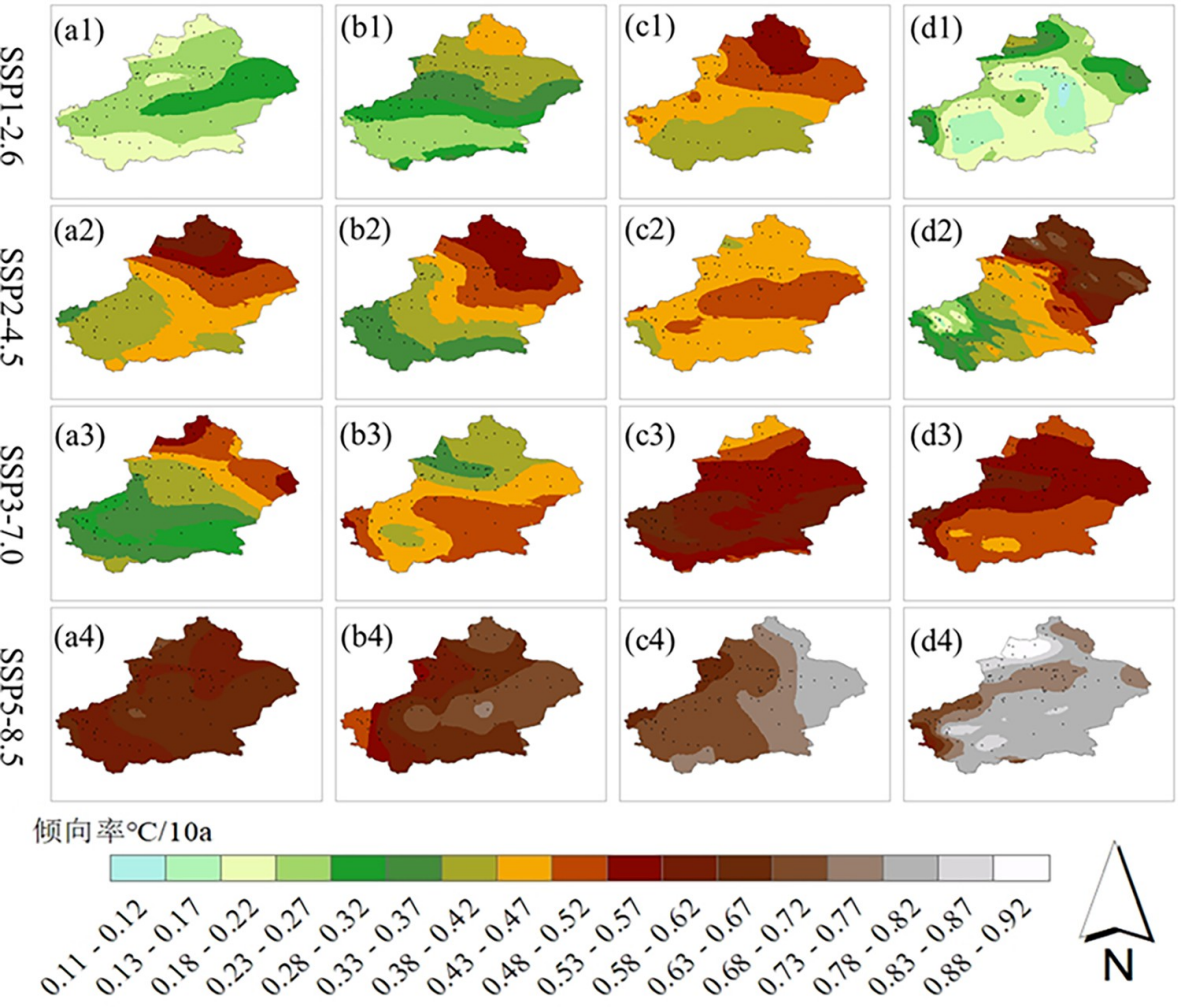
Spatial distribution of seasonal mean temperature in Xinjiang from 2021 to 2050.

At each site in the study area, a linear trend test was conducted for the seasonal mean temperatures from 2021 to 2050 for each of the four scenarios. The results were interpolated by kriging interpolation to obtain the spatial distributions of the seasonal mean temperature trends (Fig 7). The average rates of warming for all seasons in the future period gradually increase for scenario with increasing emissions.

In the SSP1-2.6 scenario, the rates of increase in the mean temperatures in summer and autumn become progressively greater from south to north, and the warming rates during most of spring and winter range from 0.18 to 0.32°C/10 a. In the SSP2-4.5 scenario, the mean temperature increase rates in spring, summer, and winter increase progressively from southwest to northwest; the warming rates during most of autumn range from 0.43 to 0.52°C/10 a. The warming rates in autumn range from 0.43 to 0.52°C/10 a. In the SSP3-7.0 scenario, the mean spring warming rates increase gradually from the southwest to the northwest; the warming rates over most of the region in autumn and winter range from 0.43–0.67°C/10 a. The SSP5-8.5 scenario has the highest mean winter warming rates, with warming rates that occur across most of the region ranging from 0.78 to 0.92°C/10 a.

### 3.3. Precipitation forecast for the Xinjiang region in the future period

#### 3.3.1. Trends

**Fig 8:**
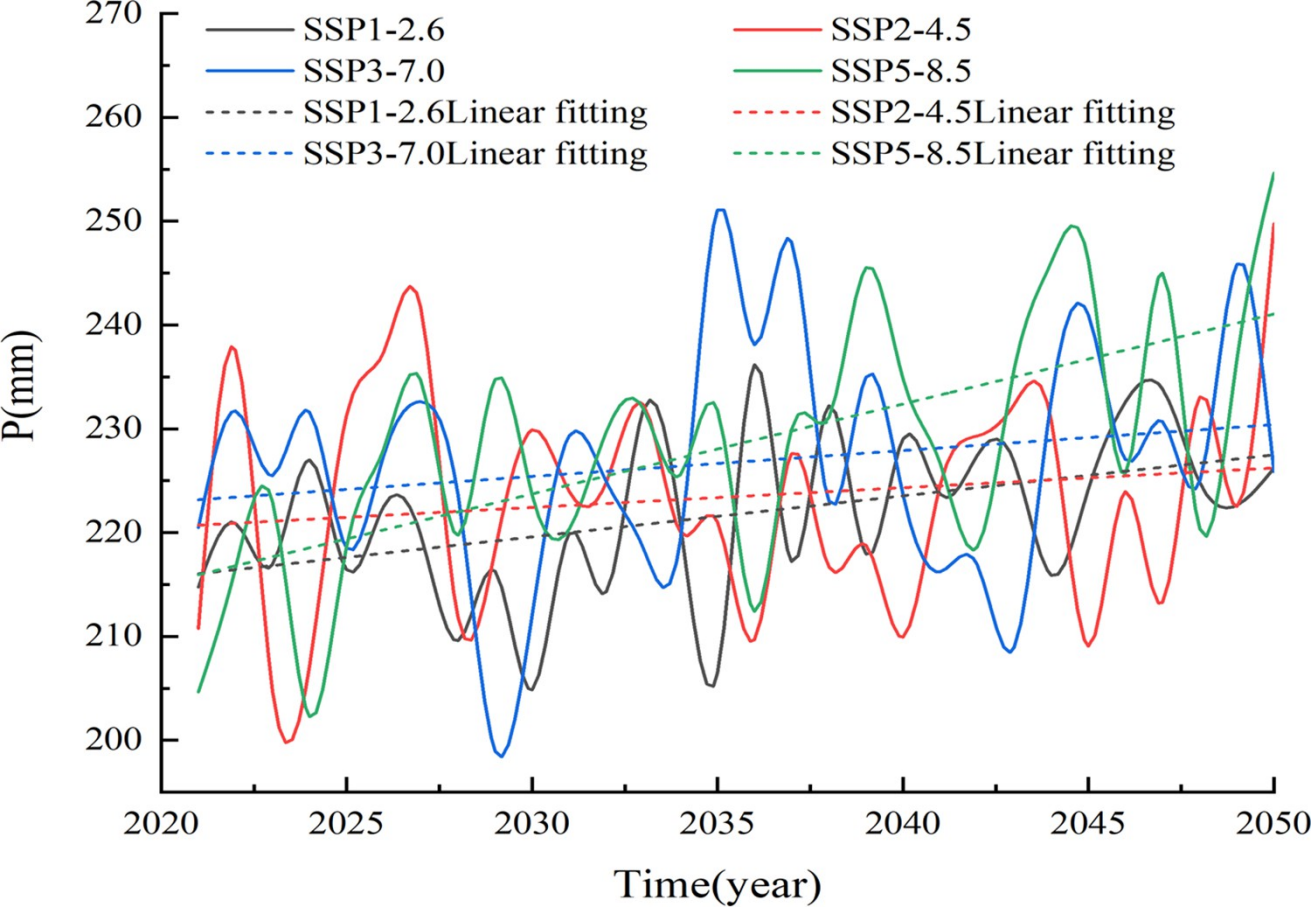
Interannual variation trend of precipitation in Xinjiang during 2021–2050.

In this study, the future precipitation data for the SSP1-2.6, SSP2-4.5, SSP3-7.0, and SSP5-8.5 scenarios in the Xinjiang region for the period from 2021 to 2050 are generated based on the results of multimodel ensemble averaging of the data from seven CMIP6 models. The future annual precipitation results for the four scenarios are shown in Fig 8. The total annual precipitation in the Xinjiang region under the four SSP-RCPs exhibited fluctuating upward trends from 2021 to 2050. The linear rates of precipitation change under the SSP1-2.6, SSP2-4.5, SSP3-7.0, and SSP5-8.5 scenarios are 3.95, 1.90, 2.50 and 8.67mm/10 a, respectively. In addition, the future precipitation amounts are significantly greater, especially under the SSP5-8.5 high-emissions scenario, where the trends in precipitation increases are greater than those under the other scenarios.

**Fig 9:**
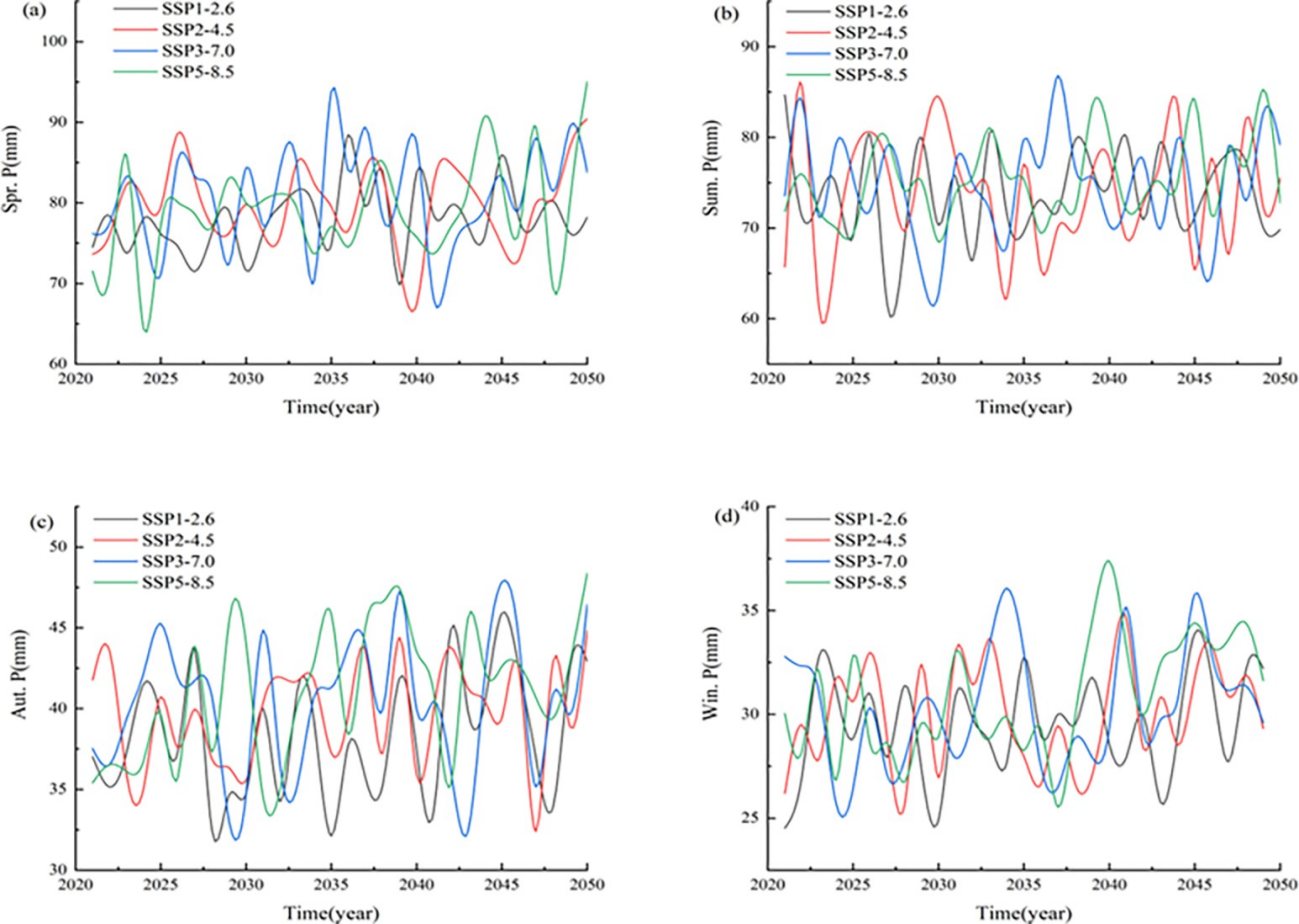
Seasonal variation trend of precipitation in Xinjiang from 2021 to 2050.

In addition, we analyzed the changes in the seasonal mean precipitation values in the Xinjiang region (Fig 9).

The results seem to show that the precipitation amounts in the Xinjiang region show increasing trends in all seasons under different scenarios, and the rates of increase vary with the scenario and season. In general, the precipitation amounts have the smallest rate of increase under the SSP2-4.5 scenario, followed by those in the SSP3-7.0 and SSP1-2.6 scenarios, and the largest rate of increase occurs under the SSP5-8.5 scenario.

In spring, the precipitation amounts increased at rates of 1.45, 0.84, 1.49, and 2.79 mm/10 a, respectively, for the four different scenarios (Table 3). In summer, the precipitation propensity rates under the different emission scenarios are 0.153, 0.285, 0.371 and 1.72 mm/10 a, respectively. In autumn, the climatic propensity rates under the four scenarios are 1.3, 0.74, 0.81 and 2.34 mm/10 a, respectively. In winter, the precipitation amounts under the four scenarios exhibited continuous increasing trends of 0.56, 0.60, 0.73 and 1.79 mm/10 a. Overall, the precipitation amounts modeled under the different scenarios increased at the slowest rates in winter and at faster rates in spring.

**Table 3:** Seasonal and interannual precipitation trends under four scenarios in Xinjiang during 2021–2050.

Trend (mm/10a)	Spring	Summer	Autumn	Winter	Interannual
SSP1-2.6	1.449	0.153	1.302	0.559	3.950
SSP2-4.5	0.835	0.285	0.735	0.597	1.899
SSP3-7.0	1.490	0.371	0.806	0.734	2.495
SSP5-8.5	2.794	1.721	2.337	1.793	8.66

#### 3.3.2. Spatial distribution

**Fig 10:**
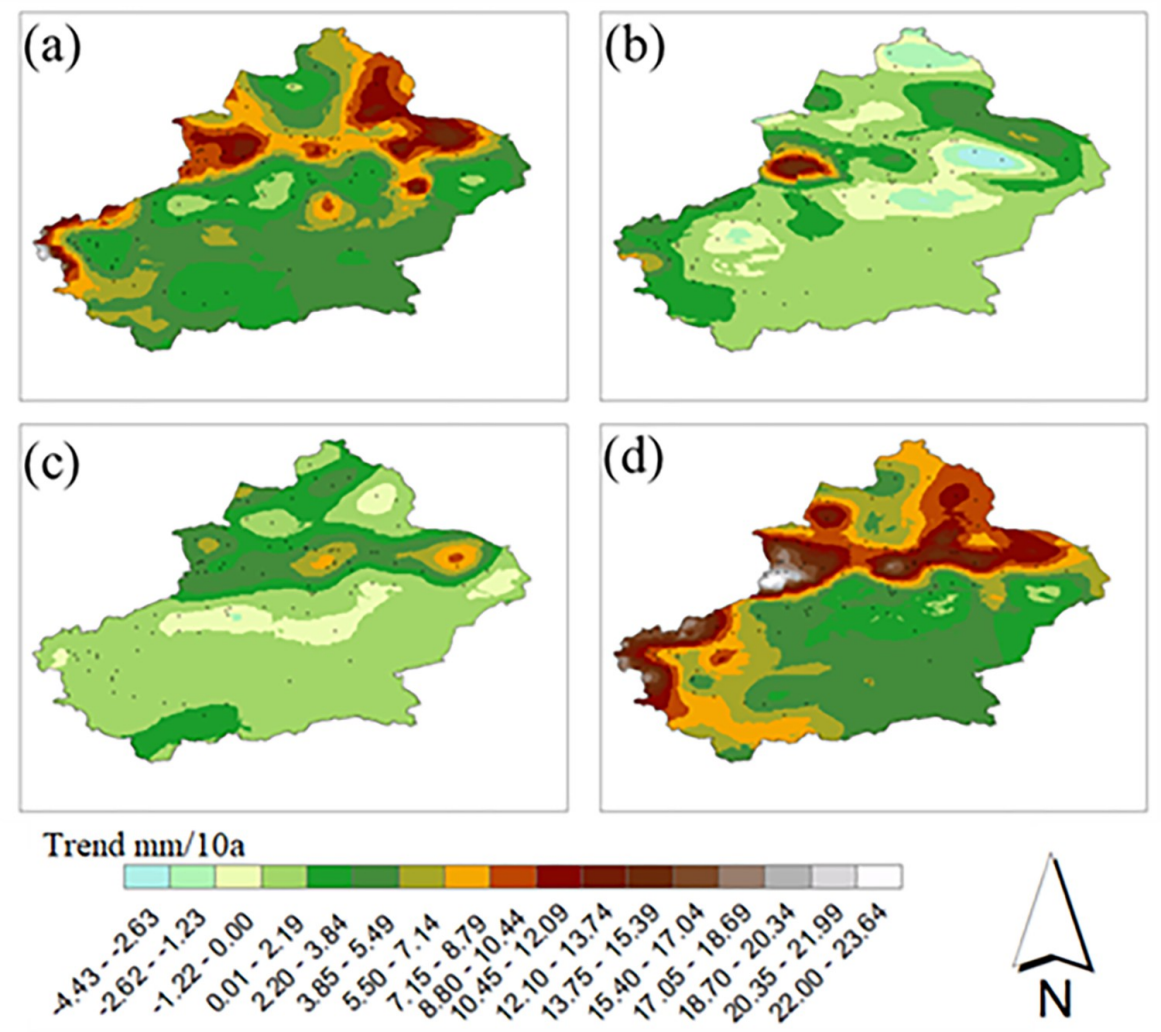
Spatial distribution of future mean precipitation trend in Xinjiang. (a)SSP1-2.6、(b)SSP2-4.5、(c)SSP3-7.0、(d)SSP5-8.5.

Moreover, we analyzed the spatial distributions of the trends in the annual average precipitation changes in the future period (2021–2050) (Fig 10), and the trends in the annual precipitation changes under the different scenarios were quite different. The results show that the precipitation amounts in the whole Xinjiang region under all four scenarios show increasing trends.

Under the SSP1-2.6 scenario, the areas with the largest increasing precipitation trends are mainly concentrated in the eastern parts of Altay, Yili, Tacheng, and Changji; most of the areas south of the Tianshan Mountains have increasing precipitation rates that range from 2.20–5.49 mm/10 a. Under the SSP2-4.5 scenario, the areas with the largest increasing precipitation trends are located mainly in the southern part of Yili, and decreasing precipitation trends occur in the northern parts of Altay, Turfan, and Hami. Under the SSP3-7.0 scenario, the rates of precipitation increases in most areas of Xinjiang were between 0 and 5.49 mm/10 a, and the rates of increase in the annual precipitation amounts gradually increased from south to north; the area with the largest increased precipitation trends was present mainly in Barkun, where the rates of increase were between 8.80 and 10.44 mm/10a. Under the SSP5-8.5 scenario, the rates of increase for the annual precipitation amounts gradually increase from the southeast to the northwest, and the rates of precipitation increase gradually increase from the southeast to the northwest. Under the SSP5-8.5 scenario, the rates of increase for the annual precipitation amounts gradually increase from the southeast to the north; the areas with trends showing greater precipitation increases are mainly located in northern and southwestern Xinjiang, and the rates of precipitation increase in the vast majority of southern Xinjiang range from 2.2 to 7.14 mm/10 a.

A comparison of the four scenarios reveals that, overall, the SSP5-8.5 scenario yields the largest precipitation increase in Xinjiang, followed by the SSP1-2.6 scenario and the SSP3-7.0 and SSP2-4.5 scenarios, which reveal smaller increases and smaller differences in the overall precipitation increases in Xinjiang.

**Fig 11:**
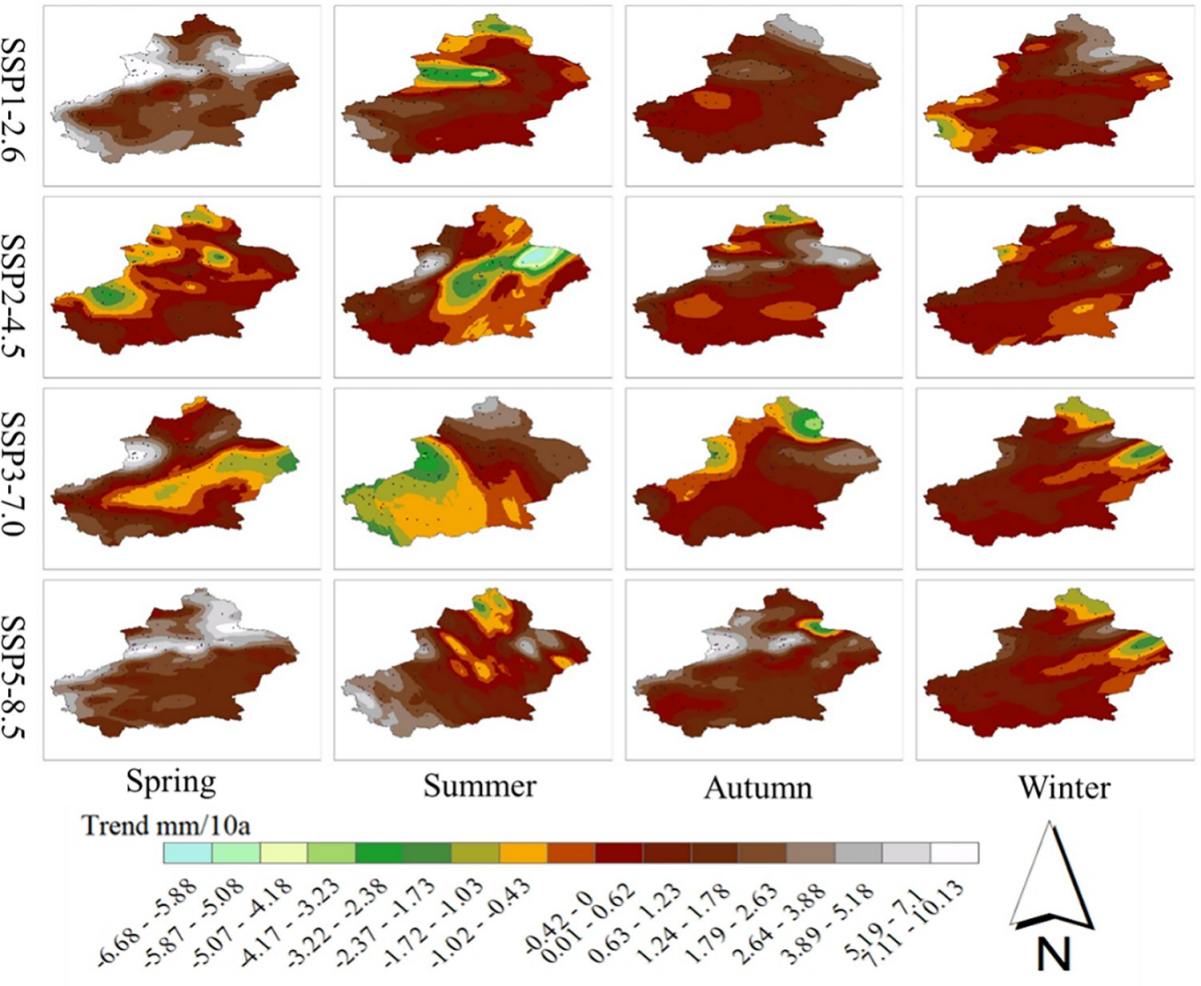
Spatial distribution of seasonal precipitation trends in Xinjiang from 2021 to 2050.

Trend tests were performed on the seasonal mean precipitation values under the four scenarios for the period from 2021 to 2050 at each site in the study area, and the results obtained were interpolated using the kriging interpolation method to obtain the spatial distributions of the seasonal mean precipitation trends (Fig 11). Overall, the rates of precipitation increase for all seasons in the future period gradually increase as the emission scenarios increase, except for SSP1-2.6.

In the SSP1-2.6 scenario, the rates of increase in the mean precipitation in autumn and winter increase gradually from southwest to northeast, with the rates of increase in most areas ranging from 0–3.88 mm/10a. In the SSP2-4.5 scenario, the rates of increase in the mean precipitation in autumn and winter both increase gradually from south to north, with the rates of precipitation increase in most areas in autumn ranging from 0.43–0.52 mm/10 a. Under the SSP3-7.0 scenario, the rates of increase in the mean precipitation in spring gradually increase from southwest to northwest; the rates of precipitation increase in autumn and winter range from 0.43 to 0.67 mm/10 a in most areas; the rates of increase in the mean precipitation in spring are the largest under the SSP5-8.5 scenario; and the rates of increase in the mean precipitation in most areas near the northern border range from 3.89 to 10.13 mm/10 a; however, in the vast majority of areas near the southern border, the rates of increase are between 3.89 and 10.13 mm/10 a. The average rates of precipitation increase were between 3.89 and 10.13 mm/10 a along most of the northern border, while the average rates of precipitation increase were between 0.63 and 2.63 mm/10a along most of the southern border.

## 4. Discussion

In this paper, climate change in the Xinjiang region from 2021 to 2050 were analyzed by using existing historical climate data and future climate data predicted by the model. This paper distinguishes between interannual and seasonal periods, which can reflect the characteristics of the changes at different time scales. Spatial analyses were carried out using bilinear interpolation and kriging interpolation methods and thus can better represent the spatial differences in climate change. The CMIP6 model predicts the future temperature and precipitation values in the Xinjiang region with high degrees of uncertainty. Several models that are currently in use simulate temperatures better than precipitation, and the use of multimodal model averaging can better simulate the precipitation amounts.

The temperature and precipitation in the Xinjiang region in all seasons under the different scenarios exhibited increasing trends, and the growth rates varied with the scenario and season. The annual mean temperature increases slightly at rates of 0.32, 0.46, 0.47, and 0.67°C/10 a under the four scenarios, respectively, and the annual mean precipitation changes at rates of 3.95, 1.90, 2.50, and 8.67 mm/10a under the four scenarios, respectively. In summary, the climate of the Xinjiang region will be characterized by warming and humidification from 2021 to 2050, and the overall rates of increase in temperature and precipitation will increase with increasing radiative emissions. These results are consistent with the findings of Li et al [35–37]. In the future, there will be significant increases in temperature and precipitation in the northwestern region of China, and there will be a warming and humidification trend in Xinjiang.

The simulation results of the seven CMIP6 models for the temperatures in the Xinjiang region showed very small deviations from the observations, but the simulation results for the precipitation amounts showed large deviations, all of which overestimated the precipitation in the Xinjiang region, which may lead to underestimations of future drought in the Xinjiang region, China, in this study. Future studies need to use additional climate models, and different multimodel ensemble methods [38,39], such as linear regression [40] and Bayesian averaging [41,42], can also be used to improve the credibility of the results. In this study, the delta method was used for statistical downscaling, and other methods of statistical downscaling and dynamical downscaling will be further combined in the future to explore the applicability and limitations of various downscaling methods in the Xinjiang region. In this paper, only simulations of future temperature and precipitation were carried out, after which climate, ecological and hydrological simulations can be carried out based on the output of the climate system model.

## 5. Conclusion

In this paper, seven global climate models were selected from CMIP6, and the temperature and precipitation data from 1961 to 2014 were processed via delta downscaling and bias correction. Multimodel ensemble averaging was subsequently applied to predict the spatial and temporal changes in temperature and precipitation in the Xinjiang region from 2021 to 2050 under four different climate scenarios, and the main conclusions are as follows:

1. The mean temperature and precipitation values that are expressed by the multimodel ensemble averages from 1961 to 2014 and the trends of the changes are in good agreement with the observed data, and the temperature and precipitation predictions for the Xinjiang region from 2021 to 2050 under different scenarios have a high degree of confidence.
2. The annual mean temperatures in the Xinjiang region from 2021 to 2050 increase slightly at rates of 0.32, 0.46, 0.47, and 0.67°C/10 a under the four scenarios, respectively. The higher the radiative forcing scenario is, the greater the rate of increase in seasonal temperature; the rate of temperature increase over the seasons is ranked as autumn > summer > spring > winter.
  Spatially, the rates of increase in the annual mean temperatures under the SSP1-2.6 scenario gradually increase from south to north, and the rates of increase in the annual mean temperatures for the SSP2-4.5 scenario gradually increase from southwest to northwest. The rates of temperature increase in the vast majority of Xinjiang Province under the SSP3-7.0 scenario range from 0.47–0.51°C/10 a. The rates of temperature increase in most parts of Xinjiang under the SSP5-8.5 scenario range from 0.67–0.72°C/10 a. As the emission increases, the average warming rates in all seasons gradually increase in the future.
3. From 2021 to 2050, the average annual precipitation in Xinjiang will change at rates of 3.95, 1.90, 2.50, and 8.67 mm/10 a under the four SSPs. The precipitation amounts predicted under the different scenarios increase at the slowest rates in the winter and at faster rates in the spring.
  Spatially, the four scenarios show increasing precipitation trends across the whole Xinjiang region. The SSP1-2.6 scenario revealed that the areas with greater increasing precipitation trends are mainly concentrated in the eastern parts of Altay, Yili, Tacheng, and Changji. The SSP2-4.5 scenario revealed that the area with the largest increasing precipitation trend is mainly in the southern part of Yili. The SSP3-7.0 scenario shows that the rates of increase in annual precipitation gradually increase from the south to the north. Under the SSP5-8.5 scenario, the rates of increase in annual precipitation become progressively greater from southeast to west to north. Overall, the rates of precipitation increase for all seasons in the future period gradually increase as the emission scenarios increase, except for SSP1-2.6.
